# Genetic variants of genes in the Notch signaling pathway predict overall survival of non-small cell lung cancer patients in the PLCO study

**DOI:** 10.18632/oncotarget.11436

**Published:** 2016-08-20

**Authors:** Yinghui Xu, Yanru Wang, Hongliang Liu, Xiaozheng Kang, Wei Li, Qingyi Wei

**Affiliations:** ^1^ Cancer Center, The First Hospital of Jilin University, Changchun, Jilin 130021, China; ^2^ Duke Cancer Institute, Duke University Medical Center, Durham, NC 27710, USA; ^3^ Department of Medicine, Duke University School of Medicine, Durham, NC 27710, USA; ^4^ Key Laboratory of Carcinogenesis and Translational Research, Ministry of Education, Department of Thoracic Surgery I, Peking University Cancer Hospital and Institute, Beijing 100142, China

**Keywords:** lung cancer, GWAS, Notch pathway, overall survival (OS), single nucleotide polymorphism (SNP)

## Abstract

The Notch signaling pathway has been shown to have biological significance and therapeutic application in non-small cell lung cancer (NSCLC). We hypothesize that genetic variants of genes in the Notch signaling pathway are associated with overall survival (OS) of NSCLC patients. To test this hypothesis, we performed multivariate Cox proportional hazards regression analysis to evaluate associations of 19,571 single nucleotide polymorphisms (SNPs) in 132 Notch pathway genes with OS of 1,185 NSCLC patients available from the Prostate, Lung, Colorectal and Ovarian (PLCO) Cancer Screening Trial. We found that five potentially functional tagSNPs in four genes (i.e., *ADAM12* rs10794069 A > G, *DTX1* rs1732793 G > A, *TLE1* rs199731120 C > CA, *TLE1* rs35970494 T > TC and *E2F3* rs3806116 G > T) were associated with a poor OS, with a variant-allele attributed hazards ratio (HR) of 1.27 [95% confidence interval (95% CI) = 1.13–1.42, *P* = 3.62E-05], 1.30 (1.14–1.49, 8.16E-05), 1.40 (1.16–1.68, 3.47E-04), 1.27 (1.11–1.44, 3.38E-04), and 1.21 (1.09–1.33, 2.56E-04), respectively. Combined analysis of these five risk genotypes revealed that the genetic score 0–5 was associated with the adjusted HR in a dose-response manner (*P*_trend_ = 3.44E-13); individuals with 2–5 risk genotypes had an adjusted HR of 1.56 (1.34–1.82, 1.46E-08), compared with those with 0–1 risk genotypes. Larger studies are needed to validate our findings.

## INTRODUCTION

Globally, lung cancer was the most common cancer and the leading cause of cancer-related deaths, particularly for males, in 2012 [1]. While early detection and new therapies have increased overall survival (OS) of most cancers, survival of patients with lung cancer has not been significantly improved, with a 5-year survival of 18% [2]. This low survival rate is partly because more than one-half of cases have been diagnosed at a late stage, with an even lower 5-year survival rate of 4% [2]. Non-small cell lung cancer (NSCLC) is the most frequent type, accounting for over 85% of all the cases, with a predicted 5-year survival rate of about 15.9% [3].

Prognostic assessment is important for the NSCLC therapeutic choice. Tumor- and patient-related factors are two major factors that are associated with OS of NSCLC patients [4]; the former includes primary site, histology and tumor stage, and the latter includes age, sex, performance status and environmental factors, such as nutrition and choice of treatment. Besides, many prognostic molecular markers have also been assessed in NSCLC. For example, expression levels of or mutations in *ERCC1* [5], *RRM1* [6] and *BRCA1* [7] have been shown to have a predictive value for selection of patients who will benefit from platinum-based chemotherapy. Meanwhile, some drugs like gefitinib and crizotinib targeting *EGFR* and *EML4-ALK* mutations are recommended for patients with sensitizing mutations on the basis of an observed superior response rate, longer progression-free survival and better toxicity tolerance [4]. However, these factors remain insufficient at the personal level to determine the most appropriate therapeutic choice and clinical outcomes. Recently, genetic factors are considered to play an important role in lung cancer susceptibility [8] and prognosis [9–12]. Furthermore, it is estimated that genetic factors account for 20–95% of the variability in anti-cancer effects and toxicities [13]. Therefore, it is essential to identify the role of some genetic factors in lung cancer prognosis, which could lead to a comprehensive prognostic model for NSCLC.

The Notch signaling pathway regulates cell-cell communication, which involves gene regulation mechanisms that control cell proliferation, differentiation and apoptosis processes [14]. There are four receptors (Notch 1–4), five ligands (Delta-like 1, Delta-like 3, Delta-like 4, Jagged-1 and Jagged-2) and some downstream components in the Notch signaling pathway that can be activated by Notch ligands binding to their receptors. The combination of the ligands and the receptors can lead to translocation of the Notch intracellular domain (ICD) to the nucleus. Then, under the function of DNA-binding protein and transcriptional activators, ICD can activate the transcription of downstream helix-loop-helix (HLH) family genes, which can act as transcriptional repressors to inhibit cell differentiation process. Deregulation of the receptors or ligands involved in this pathway has been reported to be associated with pathogenesis of many human hematological malignancies and solid tumors [15]. High expression levels of *NOTCH1* and *NOTCH2* mRNA were found to be significantly associated with OS of ovarian cancer patients [16], and a 10-gene signature (*FZD4*, *HES1*, *PSEN2*, *JAG2*, *PPARG*, *FOS*, *HEY1*, *CDC16*, *MFNG*, and *EP300*) of the Notch pathway was identified to be associated with a high risk of recurrence of ovarian cancer [17]. Also, *NOTCH1* overexpression might predict poorer survival and more aggressive behavior in patients with hepatocellular carcinoma [18]. In addition, Delta-like 4 (DLL4) and Jagged-1(JAG1) were reported to be involved in the process of tumor angiogenesis [19]. For NSCLC, high expression levels of *NOTCH1* and *NOTCH3* have been found to be significantly associated with poor prognosis in lung adenocarcinoma [20, 21], whereas *DLL4* and *HES1* were also positively associated with poor OS of NSCLC patients [22], suggesting that Notch signal has biological significance and therapeutic application in NSCLC.

To date, there are no reported studies using large-scale genome-wide association study (GWAS) datasets to investigate the role of genetic variants of genes in the Notch pathway in NSCLC survival. Therefore, we hypothesize that genetic variants in the Notch signaling pathway genes are associated with OS of NSCLC patients.

## RESULTS

### Multivariate analyses of associations between SNPs and NSCLC OS

**Figure 1:**
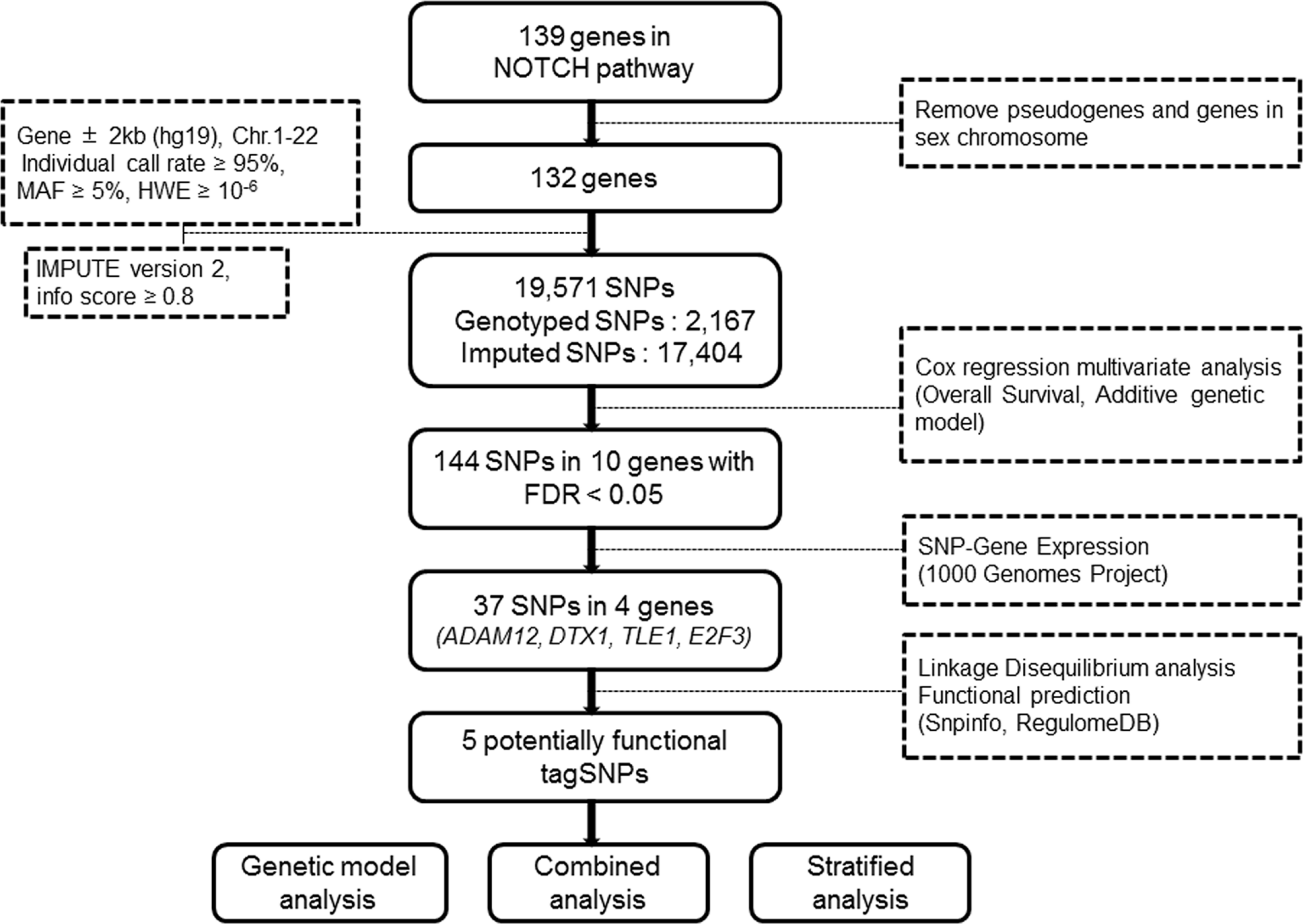
Research workflow chart

**Figure 2:**
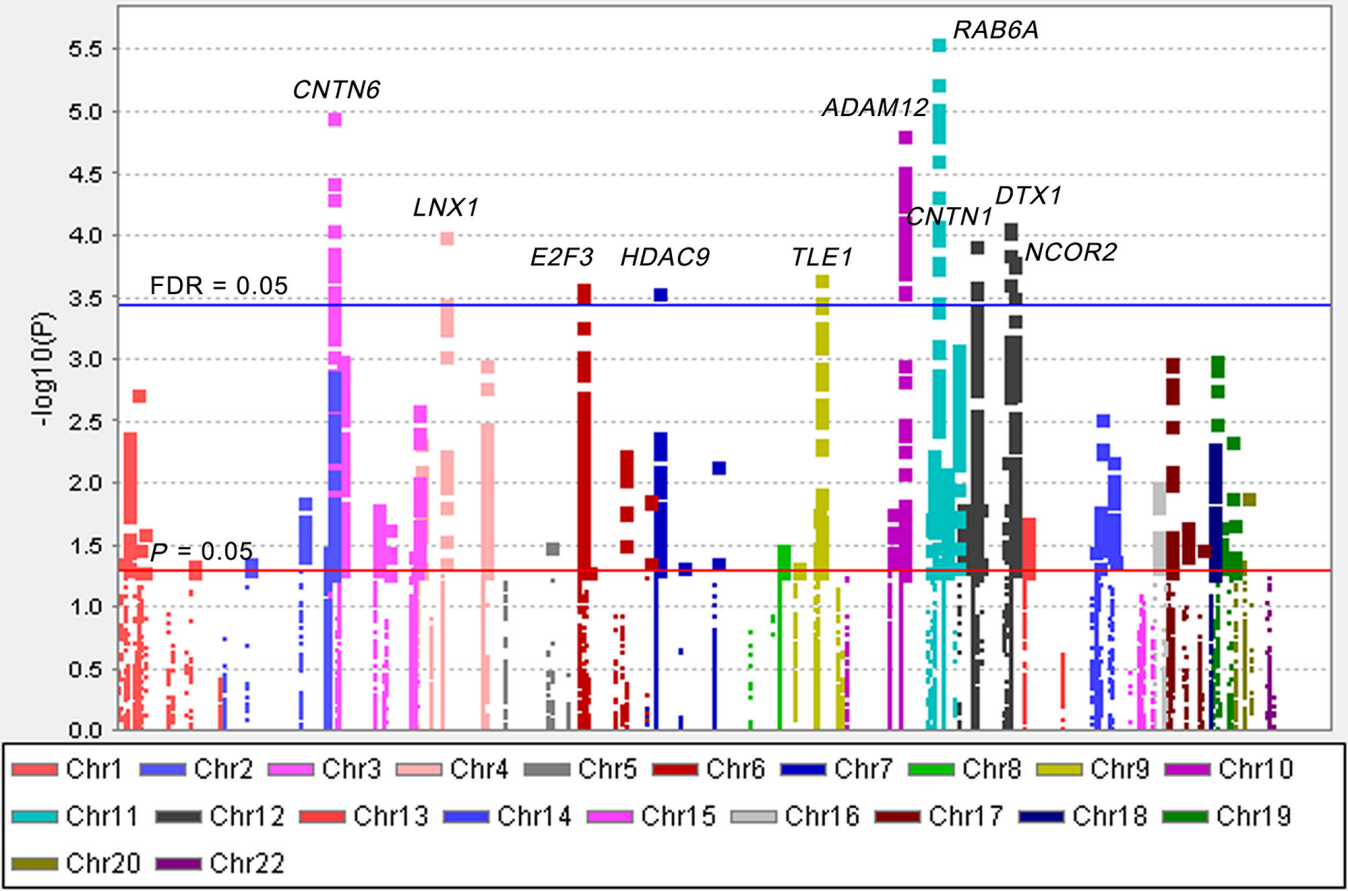
Manhattan plot of 19,571 SNPs of Notch pathway genes in the PLCO study The statistical values across the autosomes for associations between 19,571 SNPs and overall survival are plotted as −log10 *P* values. The red horizontal line indicates *P* = 0.05 and the blue line indicates FDR = 0.05.

The overall workflow of the present study is shown in Figure 1. Basic characteristics of 1,185 NSCLC patients from the PLCO study were described previously (Supplementary Table S1) [23]. We first performed multivariate Cox proportional hazards regression analysis to evaluate associations between 19,571 SNPs (i.e., 2,167 genotyped and 17,404 imputed SNPs) of the Notch signaling pathway genes (Supplementary Table S2) and NSCLC OS with adjustment of age, sex, smoking status, histology, tumor stage, chemotherapy, radiotherapy and surgery. Among of these SNPs, 2,103 SNPs were individually significantly associated with OS at *P* < 0.05 in an additive genetic model. After the corrections for multiple testing, 144 SNPs in 10 genes (*ADAM12*, *CNTN1*, *CNTN6*, *DTX1*, *HDAC9*, *LNX1*, *NCOR2*, *RAB6A*, *TLE1* and *E2F3*) with false discovery rate (FDR) < 0.05 were selected for further analyses (Figure 2).

### Functional SNPs selection

**Figure 3:**
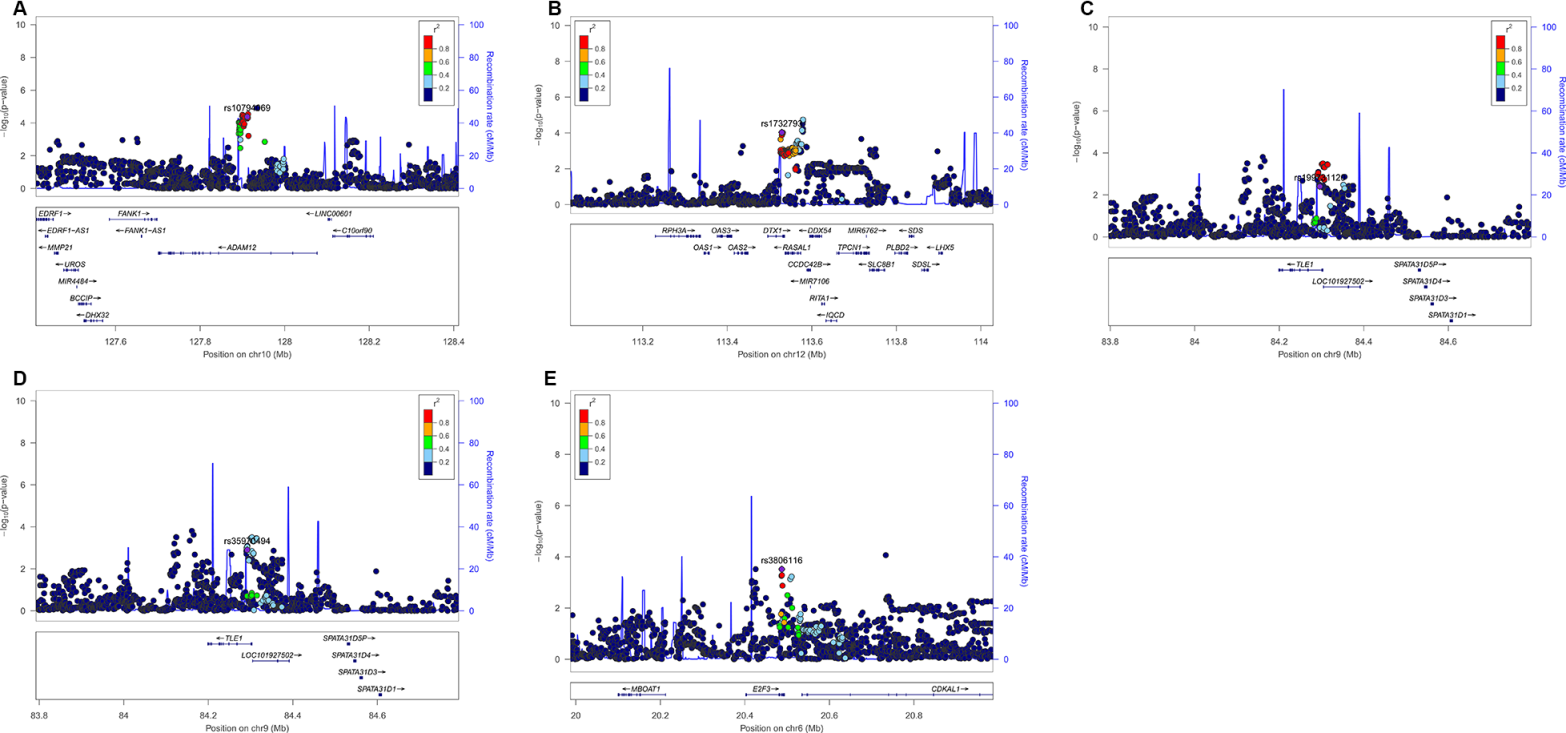
Regional association plots of the five tagSNPs The left-hand Y-axis shows the -log10 transformation of *P-value* of individual SNPs, which is plotted against the chromosomal base-pair position with an expansion of 500 KB in the flanks of the gene region. The right-hand Y-axis shows the recombination rate estimated for European populations from HapMap Data Rel 22/phase II. (**A**) *ADAM12* rs10794069; (**B**) *DTX1* rs1732793; (**C**) *TLE1* rs199731120; (**D**) *TLE1* rs35970494; (**E**) *E2F3* rs3806116.

**Figure 4:**
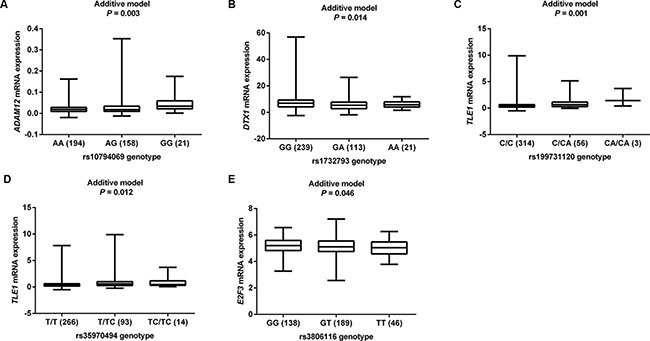
eQTL analyses of tagSNPs and corresponding gene mRNA expression All the data were from 373 individuals of European descendants from 1000 Genomes Project. (**A**) *ADAM12* rs10794069; (**B**) *DTX1* rs1732793; (**C**) *TLE1* rs199731120; (**D**) *TLE1* rs35970494; (**E**) *E2F3* rs3806116.

We then used the expression quantitative trait loci (eQTL) analyses to identify SNPs that were associated with mRNA expression levels of the corresponding genes. Of the 144 SNPs, 37 SNPs in four genes (*ADAM12*, *DTX1*, *TLE1 and E2F3*) with positive eQTL results were selected (*P*_additive_ < 0.05) (Supplementary Table S3). We then performed pairwise linkage disequilibrium (LD) analyses of the SNPs in *ADAM12*, *DTX1* and *TLE1* (only one SNP in *E2F3*) (Supplementary Figure S1). In *ADAM12*, the 24 SNPs were in high LD (all *r*^2^ > 0.8). In *DTX1*, there were moderate to high LD (*r*^2^ = 0.7–1.0) between the eight SNPs. For the four SNPs in *TLE1*, there was a high LD (*r*^2^ > 0.8) among rs199731120, rs72747302 and rs141894076, except for rs35970494 that had a low LD with the other three SNPs (*r*^2^ < 0.4). As a result, we chose five tagSNPs (i.e., rs10794069 in *ADAM12*, rs1732793 in *DTX1*, rs199731120 and rs35970494 in *TLE1*, and rs3806116 in *E2F3*) as the tagSNPs, based on the comprehensive results of *P value* and functional prediction (Snpinfo and RegulomeDB) (Table 1). All genotyped and imputed SNPs are shown in the regional association plots with an expansion of 500 KB in the flanks of the gene region, in which the selected five tagSNPs, as shown on the top of the plots, are each labeled in purple (Figure 3). Their physical locations on the genes are summarized in Supplementary Figure S2. As shown in Figure 4, we found that rs10794069 GG, rs199731120 CA/CA, and rs35970494 TC/TC genotypes were associated with increased levels of the corresponding mRNA expression (*P*_additive_ = 0.003, 0.001 and 0.012, respectively) (Figure 4A, 4C and 4D), whereas rs1732793 AA and rs3806116 TT genotypes were found to be associated with decreased mRNA expression levels (*P*_additive_ = 0.014 and 0.046, respectively) (Figure 4B and 4E).

**Table 1:** Summary of the five identified functional tagSNPs

SNP	Chr.	Position (hg19)	Gene	Location	Allele^a^	MAF	Frequency^b^		Overall Survival			SNPinfo^d^	Regulome DB^e^
							All	Death	HR (95% CI)	*P*^c^	FDR		
rs10794069	10	127911533	*ADAM12*	intron	A/G	0.25	671/437/77	437/307/54	1.27 (1.13–1.42)	3.62E-05	0.015	-	4
rs1732793	12	113528932	*DTX1*	intron	G/A	0.18	788/362/35	514/260/24	1.30 (1.14–1.49)	8.16E-05	0.023	TFBS	3a
rs199731120	9	84295857	*TLE1*	intron	C/CA	0.08	993/165/6	656/121/5	1.40 (1.16–1.68)	3.47E-04	0.047	-	3b
rs35970494	9	84292151	*TLE1*	intron	T/TC	0.18	759/340/37	497/234/29	1.27 (1.11–1.44)	3.38E-04	0.046	-	6
rs3806116	6	20487250	*E2F3*	intron	G/T	0.38	447/557/175	288/378/127	1.21 (1.09–1.33)	2.56E-04	0.042	-	-

Abbreviations: HR, hazards ratio; CI, confidence interval; FDR, false discovery rate; Chr., chromosome; MAF, minor allele frequency; TFBS, transcription factor binding sites.

a Reference/effect allele.

b Major homozygote/heterozygote/rare homozygote.

c Multivariate Cox regression analyses were adjusted for age, sex, smoking status, histology, tumor stage, chemotherapy, radiotherapy, and surgery.

d http://snpinfo.niehs.nih.gov/.

e http://regulome.stanford.edu/.

### Five potentially functional tagSNPs and survival in NSCLC patients

Then, we performed survival analyses with different genetic models for each tagSNP using univariate and multivariate Cox regression analysis methods. As shown in Table 1, we found that under an additive genetic model, *ADAM12* rs10794069 G, *DTX1* rs1732793 A, *TLE1* rs199731120 CA, *TLE1* rs35970494 TC and *E2F3* rs3806116 T variant alleles were associated with a poor NSCLC OS, with a variant-allele attributed hazard ratio (HR) of 1.27 [95% Confidence interval (95% CI) = 1.13–1.42, *P* = 3.62E-05], 1.30 (95% CI = 1.14–1.49, *P* = 8.16E-05), 1.40 (95% CI = 1.16–1.68, *P* = 3.47E-04), 1.27 (95% CI = 1.11–1.44, *P* = 3.38E-04), and 1.21 (95% CI = 1.09–1.33, *P* = 2.56E-04), respectively.

**Figure 5:**
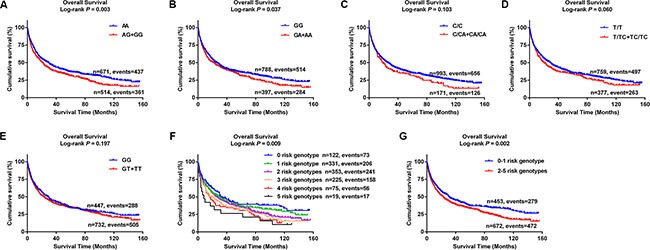
Kaplan-Meier (KM) survival curves for NSCLC patients of five tagSNPs and combined risk genotypes (**A**) *ADAM12* rs10794069; (**B**) *DTX1* rs1732793; (**C**) *TLE1* rs199731120; (**D**) *TLE1* rs35970494; (**E**) *E2F3* rs3806116; (**F**) Six groups of combined risk genotypes; (**G**) Two groups of combined risk genotypes.

Table 2 shows the results from a dominant genetic model in multivariate analyses. Compared with the corresponding common homozygous genotypes, their variant genotypes were significantly associated with a poorer OS (HR = 1.35, 95% CI = 1.17–1.56, and *P* = 3.22E-05 for rs10794069 AG+GG; 1.31, 1.13–1.51, and 4.12E-04 for rs1732793 GA+AA; 1.42, 1.17–1.72, and 4.70E-04 for rs199731120 C/CA+CA/CA; 1.31, 1.12–1.53, and 5.64E-04 for rs35970494 T/TC+TC/TC; and 1.33, 1.15–1.54, and 1.72E-04 for rs3806116 GT+TT). To provide a visual effect, we also present the Kaplan-Meier (KM) survival curves for the associations between these risk genotypes and OS in Figure 5A–5E, showing that genotypes of rs10794069 AG+GG, rs1732793 GA+AA, rs199731120 C/CA+CA/CA, rs35970494 T/TC+TC/TC and rs3806116 GT+TT were associated with a poor OS of NSCLC patients (Log-rank test: *P* = 0.003, 0.037, 0.103, 0.060 and 0.197, respectively).

**Table 2:** Associations between tagSNPs in the Notch pathway genes and overall survival of NSCLC patients

Gene/SNP	Genotype	Frequency		Univariate analysis		Multivariate analysis^a^	
		All	Death (%)	HR (95% CI)	*P*	HR (95% CI)	*P*
*ADAM12* rs10794069 A > G	AA	671	437 (65.1)	1.00		1.00	
	AG	437	307 (70.3)	1.23 (1.06–1.43)	0.006	1.33 (1.15–1.55)	1.52E-04
	GG	77	54 (70.1)	1.25 (0.95–1.66)	0.117	1.47 (1.10–1.95)	0.009
	Trend test				0.005		3.62E-05
	AG+GG	514	361 (70.2)	1.23 (1.07–1.42)	0.003	1.35 (1.17–1.56)	3.22E-05
*DTX1* rs1732793 G > A	GG	788	514 (65.2)	1.00		1.00	
	GA	362	260 (71.8)	1.19 (1.02–1.38)	0.025	1.27 (1.09–1.48)	0.002
	AA	35	24 (68.6)	0.99 (0.65–1.48)	0.942	1.90 (1.26–2.88)	0.003
	Trend test				0.092		8.16E-05
	GA+AA	397	284 (71.5)	1.17 (1.01–1.35)	0.038	1.31 (1.13–1.51)	4.12E-04
*TLE1* rs199731120 C > CA	C/C	993	656 (66.1)	1.00		1.00	
	C/CA	165	121 (73.3)	1.17 (0.96–1.42)	0.118	1.40 (1.15–1.71)	0.001
	CA/CA	6	5 (83.3)	1.29 (0.53–3.10)	0.576	1.90 (0.78–4.61)	0.156
	Trend test				0.101		3.47E-04
	C/CA+CA/CA	171	126 (73.7)	1.17 (0.97–1.42)	0.104	1.42 (1.17–1.72)	4.70E-04
*TLE1* rs35970494 T > TC	T/T	759	497 (65.5)	1.00		1.00	
	T/TC	340	234 (68.8)	1.13 (0.96–1.32)	0.134	1.29 (1.10–1.51)	0.002
	TC/TC	37	29 (78.4)	1.44 (0.99–2.10)	0.055	1.54 (1.05–2.25)	0.026
	Trend test				0.029		3.38E-04
	T/TC+TC/TC	377	263 (69.8)	1.15 (0.99–1.34)	0.060	1.31 (1.12–1.53)	5.64E-04
*E2F3* rs3806116 G > T	GG	447	288 (64.4)	1.00		1.00	
	GT	557	378 (67.9)	1.06 (0.91–1.23)	0.465	1.31 (1.12–1.53)	0.001
	TT	175	127 (72.6)	1.24 (1.01–1.53)	0.041	1.40 (1.13–1.73)	0.002
	Trend test				0.058		2.56E-04
	GT+TT	732	505 (69.0)	1.10 (0.95–1.27)	0.198	1.33 (1.15–1.54)	1.72E-04
Number of risk genotypes^b^	0	122	73 (59.8)	1.00		1.00	
	1	331	206 (62.2)	1.14 (0.87–1.49)	0.338	1.62 (1.23–2.13)	5.44E-04
	2	353	241 (68.3)	1.29 (0.99–1.67)	0.059	1.96 (1.49–2.57)	1.19E-06
	3	225	158 (70.2)	1.45 (1.10–1.92)	0.009	2.33 (1.75–3.11)	8.37E-09
	4	75	56 (74.7)	1.57 (1.11–2.23)	0.011	2.84 (1.98–4.08)	1.40E-08
	5	19	17 (89.5)	1.98 (1.17–3.35)	0.011	3.40 (1.99–5.80)	7.48E-06
	Trend test				1.05E-04		3.44E-13
	0–1	453	279 (61.6)	1.00		1.00	
	2–5	672	472 (70.2)	1.26 (1.09–1.46)	0.002	1.56 (1.34–1.81)	1.46E-08

Abbreviations: SNP, single nucleotide polymorphisms; NSCLC, non-small cell lung cancer; HR, hazards ratio; CI, confidence interval.

a Multivariate Cox regression analyses were adjusted by age, sex, smoking status, histology, tumor stage, chemotherapy, radiotherapy, and surgery.

b Risk genotypes included rs10794069 AG+GG, rs1732793 GA+AA, rs199731120 C/CA+CA/CA, rs35970494 T/TC+TC/TC, and rs3806116 GT+TT.

### Combined analyses of five tagSNPs

To evaluate the joint effect of the five tagSNPs on OS of NSCLC patients, we combined the genotypes of rs10794069 AG+GG, rs1732793 GA+AA, rs199731120 C/CA+CA/CA, rs35970494 T/TC+TC/TC and rs3806116 GT+TT (under a dominant genetic model) into a genetic score to define the combined risk genotypes. We firstly categorized all the patients into six groups: 0 to 5 risk genotypes. As a result, we found that there was a risk-genotype dose-response in the effect on OS associated with the genetic score (*P*_trend_ = 3.44E-13) after adjustment for other host and clinical covariates (Table 2). Then, we dichotomized all the patients into a low-risk group (0–1 risk genotypes) and a high-risk group (2–5 risk genotypes). A similar result was observed that the high-risk group notably had 1.56 fold increased risk of death (95% CI = 1.34–1.81, *P* = 1.46E-08), compared with the low-risk group. KM curves were also provided to illustrate the association between the number of risk genotypes (the genetic score) and NSCLC OS (Figure 5F–5G).

### Stratified analyses for the effect of combined risk genotypes on NSCLC OS

We then performed stratified analyses to evaluate whether the combined effect of risk genotypes as defined by the genetic score on NSCLC OS was affected by host and clinical characteristics, including age, sex, smoking status, histology, tumor stage, chemotherapy, radiotherapy and surgery. In the multivariate analyses, we found that patients with the high (2–5) risk genotypes showed significantly worse prognosis in most subgroups, except for never smoking group that had a much reduced number of observations (Supplementary Table S4). KM curves were also performed to clearly demonstrate the associations between the combined risk genotypes and NSCLC OS in the subgroups of each clinical characteristic (Supplementary Figure S3A–S3R). However, we found no difference between the subgroups of each clinical characteristic (*P* > 0.05) as assessed by the heterogeneity test (Supplementary Table S4).

## DISCUSSION

The Notch signaling pathway controls and regulates cell proliferation, differentiation and apoptosis processes [14], and deregulation of this pathway has been reported to be associated with the development of various cancers, including cancers of the ovary [16, 17], liver [18], prostate [24], brain [25], kidney [26], colorectum [27], skin [28] and lung [20–22]. Therefore, alternations in the Notch pathway in cancer cells are increasingly being recognized. However, to the best of our knowledge, this is the first pathway analysis using the largest GWAS datasets for associations between SNPs of Notch pathway genes and NSCLC OS.

In the present study of genetic variants of 132 genes (after removal of one pseudogene and six genes in chromosome X) in the Notch signaling pathway and NSCLC OS using the published GWAS datasets, we identified *ADAM12* rs10794069 A > G, *DTX1* rs1732793 G > A, *TLE1* rs199731120 C > CA, *TLE1* rs35970494 T > TC and *E2F3* rs3806116 G > T as predictors of NSCLC OS. Specifically, the risk alleles, rs10794069G, rs1732793A, rs199731120CA, rs35970494TC and rs3806116T as well as their combined risk genotypes were associated with a poorer OS in NSCLC patients, in a risk-genotype dose-response manner. Importantly, we found that these five SNPs were associated with their gene mRNA expression levels as well, which provides further supports for the biological plausibility of our findings. The five identified potentially functional SNPs highlighted the roles of four genes (*ADAM12*, *DTX1*, *TLE1 and E2F3*) in NSCLC patient survival. It has been well known that tumor, histology, different treatment strategies are related to prognosis of NSCLC. In the current study, we included these factors as covariates for survival analysis and also performed stratified analysis of these factors. However, no heterogeneity between subgroups was observed, and patients with the high genetic risk scores all showed an increased risk of death in each subgroup of these three factors.

*ADAM12*, located in the 10q26 chromosome region, encodes an enzyme called a disintegrin and metalloprotease 12 (ADAM12). The enzyme is a membrane-anchored protein that has been implicated in a variety of biological processes involving cell-cell and cell-matrix interactions. *ADAM12* has been reported to be associated with development and progression of many cancers. For example, its gene expression was upregulated in breast cancer tissues, compared with that of normal tissues, and high levels of the ADAM12 protein were related with poor prognosis [29]. *ADAM12* was also regarded as a new biomarker of ovarian cancer, because of its low expression levels in normal tissues and high expression levels in ovarian cancer tissues, and high expression levels were associated with a poor survival in aggressive ovarian cancer as well [30, 31]. One study suggested that the ADAM12 (uADAM12) protein was a potential non-invasive biomarker for gastric cancer due to its higher expression levels in urine samples from gastric cancer patients than that from healthy controls [32]. Other study reported that *ADAM12* was highly expressed in small cell lung cancer (SCLC) and could be an effective marker for diagnosis and prognosis [33]. Taken together, *ADAM12* acts as an oncogene that is associated with poor prognosis in many cancers. Consistent with this, in the present study, the rs10794069 GG variant genotype was associated with a poor NSCLC OS, likely by increasing *ADAM12* mRNA expression in a variant allele dose-response manner. According to the ENCODE project data from UCSC, rs10794069 is located at the DNase I hypersensitive area, where shows considerable levels of H3K4Me1 enrichment. In this region, the chromatin has lost its condensed structure after histone modifications, exposing the DNA and making it accessible to transcription factors to enhance transcriptional activity. Therefore, it is likely that SNPs in this region may influence gene expression by mediating the transcriptional activity.

*DTX1*, located in the 12q24.13 chromosome region, encodes an enzyme called Deltex 1, which is also an E3 ubiquitin ligase. Depending on the cellular context, *DTX1,* a downstream gene in the Notch pathway, either acts as an oncogene to promote the Notch pathway by activating the downstream HLH family genes to inhibit cell differentiation or acts as a suppressed gene to inhibit this pathway by forming the Notch-Deltex-Kurz protein complexes that mediate Notch receptor degradation through a ubiquitination-dependent pathway [34, 35]. Here, we propose that *DTX1* may act as a suppressed gene in NSCLC, because the rs1732793 AA variant genotype was associated with a poor NSCLC OS by decreasing *DTX1* mRNA expression. Besides, the ENCODE project data from UCSC shows a certain level of H3K4Me1 enrichment in this region, which may be associated with transcriptional activity by affecting histone modifications. Meanwhile, Snpinfo function prediction online tool confirms that rs1732793 is located in the transcriptional factor binding site (TFBS) that influences the levels or timing of the gene expression by affecting transcriptional factors binding to the specific region of DNA sequences [36].

*TLE1*, located in the 9q21.32 chromosome region, encodes a protein called transducin-like enhancer protein 1 (TLE1), which is a transcriptional co-repressor that regulates the transcriptional activity of a number of genes. *TLE1,* as a putative lung-specific oncogene, was found to be overexpressed in a subset of aggressive and advanced human lung tumors [37]. For rs199731120 in *TLE1*, it is likely a TF binding sequence, which may affect corresponding gene expression. While for rs35970494, we found no additional functional evidence. In fact, in the present study, the rs199731120 CA/CA and rs35970494 TC/TC variant genotypes were both associated with a poor OS, possibly by increasing *TLE1* mRNA expression, which is consistent with oncogenic activity of *TLE1*. However, more rigorous functional studies are needed to unravel the underlying biological mechanisms to validate our findings.

*E2F3*, located in the 6p22.3 chromosome region, encodes a transcription factor called E2F Transcription Factor 3 (E2F3), which, together with E2F1 and E2F2, constitutes the transcription activators of E2Fs family. As a potential regulatory co-factor in the Notch pathway, E2F3 is considered to participate in controlling cell cycle processes [38]. In differentiating cells, E2F1–3 function in a complex with Rb as repressors to inhibit E2F target activation and facilitate the exit from the cell cycle. The inactivation of Rb in differentiating cells resulted in a switch of E2F1–3 from repressors to activators, leading to the overactivation of E2F target genes and abnormal cell divisions [39]. In the present study, we propose that *E2F3* may have a tumor suppression effect in NSCLC, because the rs3806116 TT was a risk genotype that was associated with a poor NSCLC OS by decreasing *E2F3* mRNA expression. Except for the association with *E2F3* mRNA expression, we did not find additional functional evidence about this SNP, and more functional studies are needed to validate our findings.

There are some limitations in the present study. Firstly, it is a pathway-based analysis of a published GWAS study. We extracted the genes for the Notch signaling pathway from three major publicly recognized datasets of MSigDB website: canonical pathway, GO biological process and GO molecular function, which may have excluded some important genes of this pathway. Secondly, our findings cannot be generalized to the general population, because we just used available GWAS datasets from Caucasian populations. Thirdly, only a few clinical variables were included in the current study, while other information, such as performance, nutrition status and details of combined therapies, was not available in the PLCO dataset for further analysis. Meanwhile, the information of somatic mutations, such as those of *EGFR*, *ALK*, or *KRAS*, was also not available in the PLCO dataset for the participants recruited between 1993 and 2001. Nevertheless, according to National Comprehensive Cancer Network (NCCN) (2016.v4), the mutation frequency of these genes is relatively low (*EGFR*, 10%; *ALK*, 2–7%; *KRAS*, 5–15%) in Europeans. For additional stratification analysis, we would need a much larger sample size, perhaps an even larger sample size for analyzing the interactions between these somatic mutations and SNPs on lung cancer. Finally, we were unable to explore the biological mechanisms by which the SNPs of Notch pathway genes influence NSCLC OS, because we did not have the access to the target lung cancer tissues from the study participants.

In conclusion, we evaluated associations between genetic variants of 132 genes in the Notch signaling pathway and NSCLC OS using the PLCO GWAS dataset. We identified that *ADAM12* rs10794069, *DTX1* rs1732793, *TLE1* rs199731120, *TLE1* rs35970494 and *E2F3* rs3806116 were prognostic factors for OS in NSCLC patients. Indeed, the current study would be stronger, if the replication datasets with large sample sizes were available. In the future, we will look for both population replication and functional validation to test for our findings.

## MATERIALS AND METHODS

### Study populations

This study included 1,185 NSCLC patients participated in the Prostate, Lung, Colorectal and Ovarian (PLCO) Cancer Screening Trial, which is a randomized controlled study funded by the National Cancer Institute (NCI) [40]. For the PLCO database, a sample size of 148,000 men and women aged 55–74 from ten screening centers across the United States were enrolled between 1993 and 2001 [41]. All participants were followed for at least 13 years after enrollment [42]. The PLCO trial collected blood specimens from the first screening visit and gathered information about personal sociodemographic characteristics, family history of cancer, personal medical history, and smoking history [42]. Genomic DNA extracted from the blood samples was genotyped with Illumina HumanHap240Sv1.0, HumanHap300v1.1 and HumanHap550v3.0 (dbGaP accession: phs000093.v2.p2 and phs000336.v1.p1) [43, 44]. There were 1,185 Caucasian NSCLC patients with complete follow-up information and genotype data, which were made available in the PLCO database for survival analysis. The study protocol was reviewed and approved by the institutional review board of NCI and a written informed consent was obtained from each participant.

### Gene and SNP selection

From the Molecular Signatures Database (MsigDB), 139 genes in the Notch signaling pathway were selected, which are a collection of annotated gene sets that can be analyzed by the gene set enrichment analysis (GSEA) software (Supplementary Table S2). One pseudogene and six genes in chromosome X were removed from the gene list. Then, we performed imputation of the remaining 132 genes with IMPUTE2 according to the 1000 Genomes Project CEU data (phase 1 release V3). As a result, 19,571 SNPs in 132 genes and their ± 2 kb flanking regions were obtained with the following quality control criteria: (1) genotyping rate ≥ 95%; (2) minor allelic frequency (MAF) ≥ 0.05; and (3) Hardy-Weinberg equilibrium (HWE) ≥ 1×10^−6^. Of these 19,571 SNPs, 2,167 SNPs were available from PLCO genotyping data (dbGaP accession: phs000093.v2.p2 and phs000336.v1.p1) [43–45], and the rest of 17,404 SNPs were imputed by using the reference data of 1000 Genomes Project [46].

The eQTL [47] analysis was performed to evaluate the associations between SNPs and mRNA expression levels of their genes by using the sequencing data from lymphoblastoid cells derived from 373 individuals of European descendants from 1000 Genomes Project as mentioned earlier. LD was evaluated by using the data of the same 373 European individuals. In the present study, we identified tagSNPs by using eQTL (*P*_additive_ ≤ 0.05) and LD (r^2^ ≥ 0.6) analysis. Some other online tools, including dbSNP annotation [48], Snpinfo [36], and RegulomeDB [49], were also used to identify potentially functional SNPs.

### Statistical analysis

The NSCLC OS served as a prognostic measurement was estimated in the present study. The follow-up time was defined from NSCLC diagnosis to the last follow-up or the time of death. Associations between SNPs and OS were assessed by the multivariate Cox proportional hazards regression analysis (in an additive genetic model) with the GenABEL package of R [50] with adjustment for age, sex, smoking status, histology, tumor stage, chemotherapy, radiotherapy and surgery. Imputation was performed with IMPUTE2 according to the CEU data from 1000 Genomes project (phase 1 release V3). SNPs with info value ≥ 0.8 were used for further analysis. FDR method with a cut-off value of 0.05 was used for multiple testing corrections [51]. Kaplan-Meier curve and log-rank test were also used to estimate the effects of risk genotypes on the cumulative probability of OS. Meanwhile, the risk genotypes were summarized and combined to assess the association between the number of risk genotypes and NSCLC OS. The heterogeneity test of associations between subgroups in stratified analyses was performed by using the Chi-square-based *Q*-test.

Besides, Haploview [52] v4.2 was used to produce the Manhattan plot, and LocusZoom [53] was employed to construct the regional association plots by using the 1000 Genomes Project CEU data (phase I integrated release 3, March 2012). Linear regression analysis was applied to analyze associations between SNPs and corresponding gene expression by using PLINK 1.07. All statistical analyses were performed with SAS software (version 9.1.4; SAS Institute, Cary, NC, USA), if not specified otherwise.

## SUPPLEMENTARY MATERIALS FIGURES AND TABLES



## Abbreviations

OS: overall survival
NSCLC: non-small cell lung cancer
SNPs: single nucleotide polymorphisms
FDR: false discovery rate
eQTL: expression quantitative trait loci
LD: linkage disequilibrium
HR: hazards ratio
CI: confidence interval
ICD: intracellular domain
HLH: helix-loop-helix
GWAS: genome-wide association study
PLCO: Prostate Lung Colorectal and Ovarian Cancer Screening Trial
NCI: National Cancer Institute
MsigDB: Molecular Signatures Database
GSEA: gene set enrichment analysis
MAF: minor allelic frequency
HWE: Hardy-Weinberg Equilibrium
ROC: receiver operating characteristic curve
AUC: area under the curve
KM: Kaplan-Meier
AJCC: American Joint Committee on Cancer
ADAM12: a disintegrin and metalloprotease 12
DTX1: deltex 1
TLE1: transducin-like enhancer protein 1
NCCN: National Comprehensive Cancer Network
