# Supplementary material for: Genetic variants of genes in the Notch signaling pathway predict overall survival of non-small cell lung cancer patients in the PLCO study
Source: Oncotarget. 2016 Aug 20;7(38):61716–27. doi: 10.18632/oncotarget.11436 (PMC5308685; doi:10.18632/oncotarget.11436)
Supplement: Supplementary file 1 [file oncotarget-07-61716-s001.pdf]

Genetic variants of genes in the Notch signaling pathway predict overall survival of non-small cell lung cancer patients in the PLCO study

Supplementary Materials

SUPPLEMENTARY ACKNOWLEDGMENTS

We wish to thank all of the investigators and funding agencies that enabled the deposition of data in dbGaP and PLCO that we used in this study:

The datasets used for the analyses described in this manuscript were obtained from dbGaP at http://www.ncbi.nlm.nih.gov/gap through dbGaP accession number phs000336.v1.p1 and phs000093.v2.p2. Principal Investigators: Maria Teresa Landi. Genetic Epidemiology Branch, Division of Cancer Epidemiology and Genetics, National Cancer Institute, National Institutes of Health, Bethesda, MD, USA. Neil E. Caporaso. Genetic Epidemiology Branch, Division of Cancer Epidemiology and Genetics, National Cancer Institute, National Institutes of Health, Bethesda, MD, USA.

Funding support for the GWAS of Lung Cancer and Smoking was provided through the NIH Genes, Environment and Health Initiative [GEI] (Z01 CP 010200). The human subjects participating in the GWAS derive from The Environment and Genetics in Lung Cancer Etiology (EAGLE) case-control study and the Prostate, Lung Colon and Ovary Screening Trial and these studies are supported by intramural resources of the National Cancer Institute. Assistance with phenotype harmonization and genotype cleaning, as well as with general study coordination, was provided by the Gene Environment Association Studies, GENEVA Coordinating Center (U01HG004446). Assistance with data cleaning was provided by the National Center for Biotechnology Information. Funding support for genotyping, which was performed at the Johns Hopkins University Center for Inherited Disease Research, was provided by the NIH GEI (U01HG004438).

PLCO was also supported by the Intramural Research Program of the Division of Cancer Epidemiology and Genetics and by contracts from the Division of Cancer Prevention, National Cancer Institute, NIH, DHHS. The authors thank PLCO screening center investigators and staff, and the staff of Information Management Services Inc. and Westat Inc. Most importantly, we acknowledge trial participants for their contributions that made this study possible.

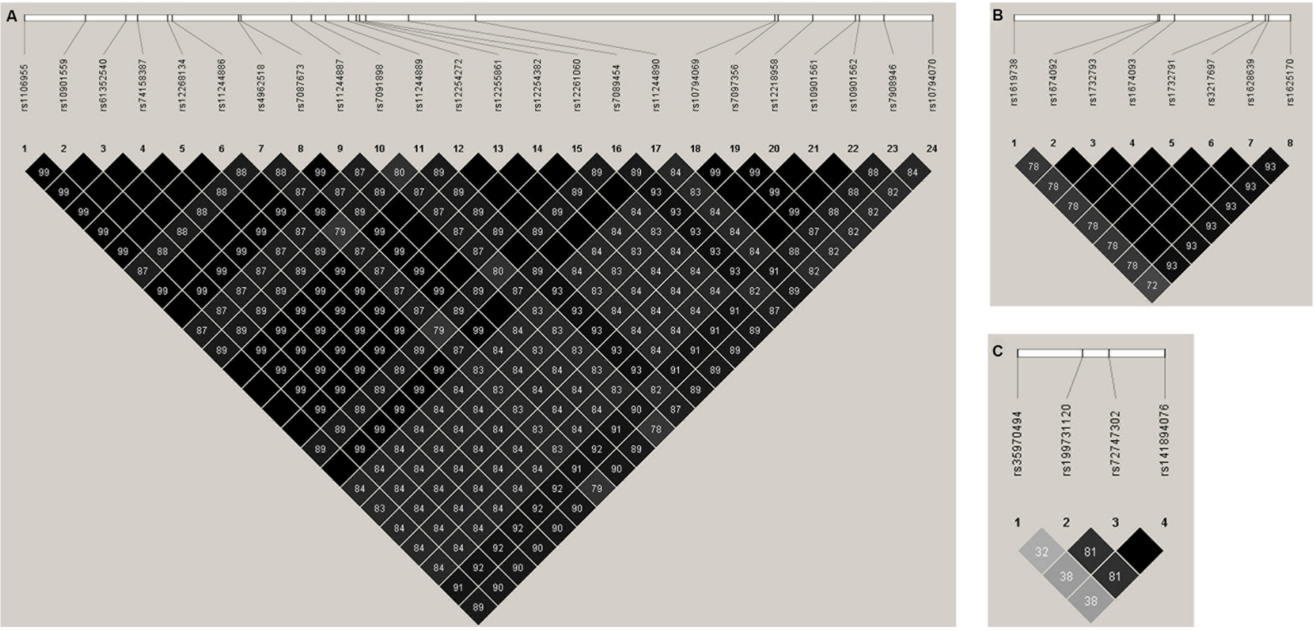

Supplementary Figure S1: Linkage disequilibrium (LD) plots for SNPs in the same gene. (**A**) the 24 SNPs in *ADAM12*; (**B**) the 8 SNPs in *DTX1*; (**C**) the 4 SNPs in *TLE1*.

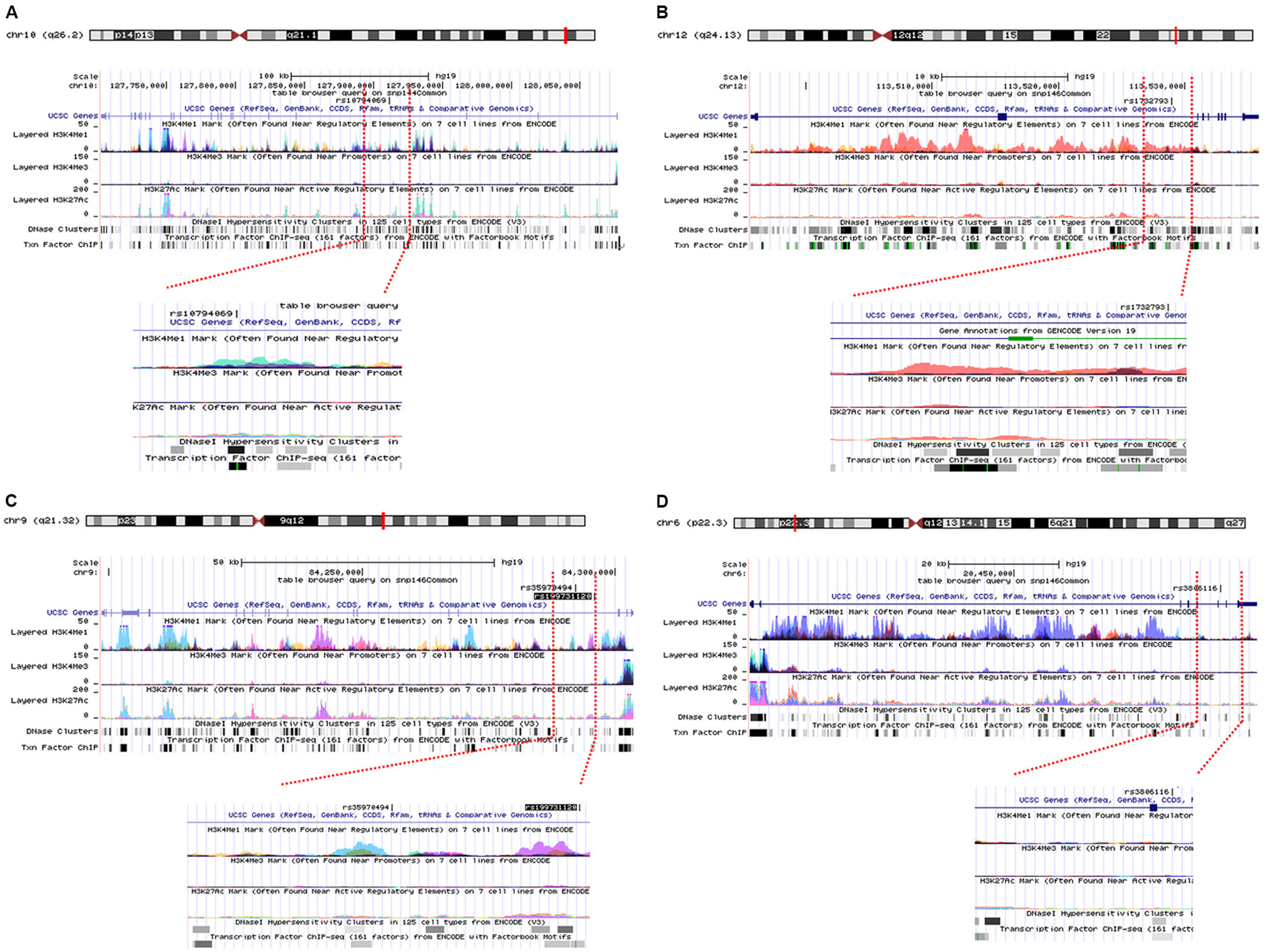

Supplementary Figure S2: ENCODE project data of tagSNPs from the University of California Santa Cruz (UCSC, http://genome.ucsc.edu/).

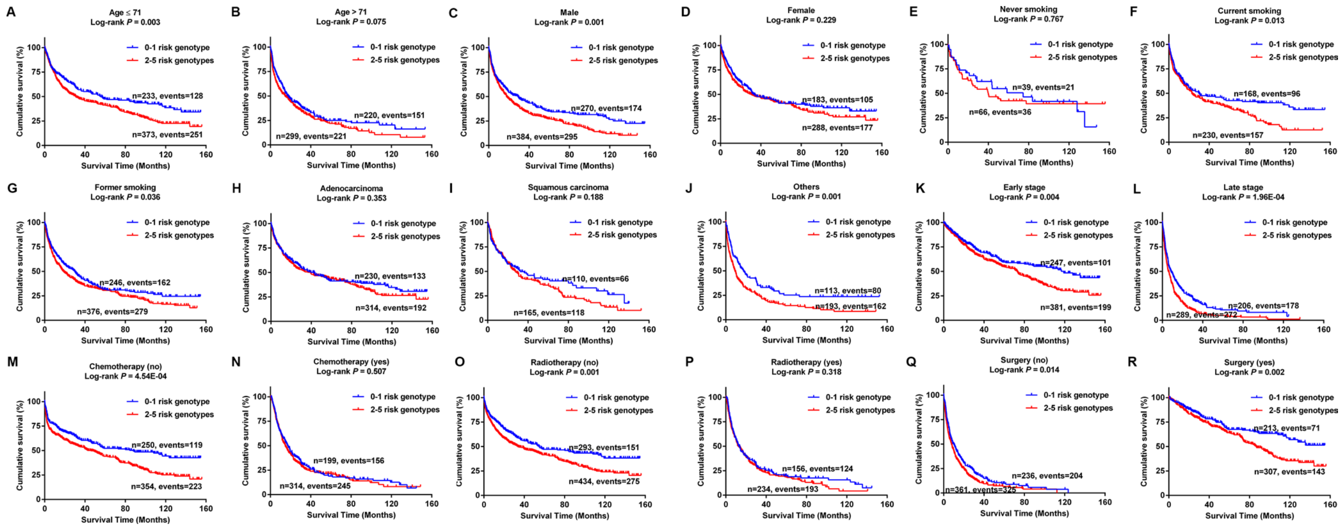

Supplementary Figure S3: Kaplan-Meier survival curves for NSCLC patients with 0–1 and 2–5 risk genotypes stratified by clinicopathological characteristics: age (A and B); sex (C and D); smoking status (E, F and G); histology (H, I and J); tumor stage (K and L); chemotherapy (M and N); radiotherapy (O and P) and surgery (Q and R).

Supplementary Table S1: Associations of demographics and clinical characteristics with OS in NSCLC patients from the PLCO study[a]

Characteristics		No.		Median Survival Time[b]	Univariate Analysis		Multivariate Analysis[c]	
		Patients	Deaths (%)		HR (95%)	*P*	HR (95%)	*P*
Total		1185	798 (67.3)	23.77				
Age[d]	≤ 71	636	400 (62.9)	36.53	1.00		1.00	
	> 71	549	398 (72.5)	14.60	1.74 (1.51–2.00)	< 0.0001	1.26 (1.09–1.46)	0.002
Sex	Male	698	507 (72.6)	19.33	1.00		1.00	
	Female	487	291 (59.8)	31.77	0.73 (0.63–0.84)	< 0.0001	0.80 (0.69–0.94)	0.005
Smoking status	Never	115	63 (54.8)	43.50	1.00		1.00	
	Current	423	272 (64.3)	23.70	1.34 (1.02–1.76)	0.038	1.79 (1.32–2.44)	0.0002
	Former	647	463 (71.6)	21.30	1.51 (1.16–1.96)	0.002	1.69 (1.26–2.27)	0.0005
Pack years[d]	≤ 49	603	399 (66.2)	23.77	1.00		1.00	
	> 49	581	398 (68.5)	23.97	1.05 (0.92–1.21)	0.458	1.01 (0.87–1.17)	0.936
	Missing	1						
Histology	Adenocarcinoma	577	348 (60.3)	36.37	1.00		1.00	
	Squamous cell carcinoma	285	192 (67.4)	29.83	1.19 (1.00–1.42)	0.057	1.14 (0.94–1.37)	0.180
	Others	323	258 (79.9)	10.87	1.89 (1.60–2.22)	< 0.0001	1.33 (1.12–1.57)	0.003
Stage	I–IIIA	655	315 (48.1)	80.13	1.00		1.00	
	IIIB–IV	528	482 (91.3)	6.87	5.08 (4.37–5.91)	< 0.0001	2.70 (2.23–3.27)	< 0.0001
	Missing	2						
Chemotherapy	No	639	367 (57.4)	53.03	1.00		1.00	
	Yes	538	423 (78.6)	14.83	1.85 (1.60–2.14)	< 0.0001	0.42 (0.35–0.50)	< 0.0001
	Missing	8						
Radiotherapy	No	762	450 (59.1)	40.50	1.00		1.00	
	Yes	415	340 (81.9)	12.33	1.94 (1.68–2.24)	< 0.0001	0.90 (0.77–1.05)	0.160
	Missing	8						
Surgery	No	637	566 (88.9)	8.27	1.00		1.00	
	Yes	540	224 (41.5)	103.33	0.15 (0.13–0.18)	< 0.0001	0.26 (0.21–0.33)	< 0.0001
	Missing	8						

Abbreviations: OS, overall survival; NSCLC, non-small cell lung cancer; PLCO, the Prostate, Lung, Colorectal and Ovarian Cancer Screening Trial; HR, hazards ratio.

[a]Reference: Yanru Wang, etc. Genetic variants in *ABCG1* predict survival of non-small cell lung cancer patients. Int J Cancer. 2016.

[b]Median survival time for overall survival.

[c]Multivariate Cox regression analyses were adjusted for all factors listed in this table.

[d]Median age is 71 years; Median pack year is 49.

Supplementary Table S2: Databases for selection of the list of genes in the Notch signaling pathway

Dataset	Name of Pathway	Genes
KEGG[a]	KEGG_NOTCH_SIGNALING_PATHWAY	47
GO[b]	GO_NOTCH_SIGNALING_PATHWAY	12
PID[c]	PID_NOTCH_PATHWAY	59
REACTOME	REACTOME_SIGNALING_BY_NOTCH	103
**TOTAL**		**139**

MSigDB database: http://www.broadinstitute.org/gsea/msigdb/.
Keyword: Notch.
Search Filters: Collection: Canonical pathways + GO gene sets; Organism: Homo sapiens. Contributor: All contributors.
[a]KEGG, Kyoto Encyclopedia of Genes and Genomes.
[b]GO, Gene Ontology.
[c]PID, Pathway Interaction Database.

Supplementary Table S3: Summary of SNPs with positive eQTL results in 373 individuals of European descendants from 1000 Genomes Project

Gene	SNP	Chr.	Location	*P*[a]	eQTL[b]	Snpinfo[c]	RegulomeDB[d]
ADAM12	rs10794069	10	intron	3.62E-05	0.003	-	4
ADAM12	rs10901559	10	intron	7.31E-05	0.013	-	6
ADAM12	rs10901561	10	intron	4.80E-05	0.003	-	5
ADAM12	rs10901562	10	intron	4.44E-05	0.003	-	5
ADAM12	rs1106955	10	intron	7.26E-05	0.014	-	5
ADAM12	rs11244886	10	intron	7.72E-05	0.013	-	5
ADAM12	rs11244887	10	intron	3.60E-05	0.014	-	5
ADAM12	rs11244890	10	intron	3.85E-05	0.014	-	6
ADAM12	rs12218958	10	intron	4.80E-05	0.003	-	4
ADAM12	rs12254272	10	intron	1.28E-04	0.014	-	-
ADAM12	rs12254382	10	intron	1.28E-04	0.014	-	-
ADAM12	rs12255861	10	intron	1.28E-04	0.014	-	-
ADAM12	rs12261060	10	intron	1.28E-04	0.014	-	-
ADAM12	rs12268134	10	intron	3.15E-05	0.013	-	5
ADAM12	rs4962518	10	intron	1.08E-04	0.037	-	-
ADAM12	rs61352540	10	intron	7.31E-05	0.013	-	6
ADAM12	rs7087673	10	intron	3.19E-05	0.013	-	6
ADAM12	rs7091898	10	intron	1.90E-04	0.036	-	3a
ADAM12	rs7097356	10	intron	5.30E-05	0.004	-	4
ADAM12	rs74158387	10	intron	3.15E-05	0.013	-	-
ADAM12	rs7908946	10	intron	2.87E-05	0.018	-	5
ADAM12	rs10794070	10	intron	9.07E-05	0.009	-	5
ADAM12	rs11244889	10	intron	1.60E-04	0.003	-	-
ADAM12	rs7089454	10	intron	1.15E-04	0.003	-	3a
DTX1	rs1619738	12	intron	2.28E-04	0.022	TFBS	4
DTX1	rs1625170	12	intron	8.04E-05	0.020	-	3a
DTX1	rs1628639	12	intron	8.82E-05	0.014	-	4
DTX1	rs1674092	12	intron	8.04E-05	0.014	TFBS	4
DTX1	rs1674093	12	intron	1.37E-04	0.014	TFBS	3a
DTX1	rs1732791	12	intron	8.04E-05	0.014	-	2b
DTX1	rs1732793	12	intron	8.16E-05	0.014	TFBS	3a
DTX1	rs3217697	12	intron	8.04E-05	0.014	-	3a
TLE1	rs141894076	9	intron	2.14E-04	6.99E-05	-	5
TLE1	rs199731120	9	intron	3.47E-04	6.52E-04	-	3b
TLE1	rs35970494	9	intron	3.38E-04	0.012	-	6
TLE1	rs72747302	9	intron	3.58E-04	6.99E-05	-	4
E2F3	rs3806116	6	intron	2.56E-04	0.046	-	-

Abbreviations: SNPs, single nucleotide polymorphisms; Chr., chromosome; eQTL, expression quantitative trait loci; TFBS, transcription factor binding sites.

[a]Multivariate Cox regression analyses were adjusted for age, sex, smoking status, histology, tumor stage, chemotherapy, radiotherapy, and surgery.

[b]eQTL results in additive model of genotypes in 373 individuals of European descendants from 1000 Genomes Project.

[c]http://snpinfo.niehs.nih.gov/snpinfo/snpfunc.htm.

[d]http://regulome.stanford.edu/.

Supplementary Table S4: Stratified association analyses of risk genotypes and OS in NSCLC patients from the PLCO study

Characteristics	0–1 risk genotype[a]		2–5 risk genotypes[a]		Univariate analysis		Multivariate analysis[b]		$P_{heterogeneity}$[c]
	All	Death (%)	All	Death (%)	HR (95%CI)	*P*	HR (95%CI)	*P*	
**Age (years)**									
≤ 71	233	128 (54.9)	373	251 (67.3)	1.38 (1.12–1.71)	0.003	1.46 (1.17–1.81)	0.001	
> 71	220	151 (68.6)	299	221 (73.9)	1.21 (0.98–1.49)	0.075	1.60 (1.29–1.98)	2.08E-05	0.557
**Sex**									
Male	270	174 (64.4)	384	295 (76.8)	1.36 (1.12–1.64)	0.002	1.54 (1.27–1.87)	1.59E-05	
Female	183	105 (57.4)	288	177 (61.5)	1.16 (0.91–1.48)	0.230	1.68 (1.30–2.17)	7.81E-05	0.595
**Smoking status**									
Never	39	21 (53.9)	66	36 (54.6)	1.09 (0.63–1.86)	0.767	1.34 (0.75–2.40)	0.322	
Current	168	96 (57.1)	230	157 (68.3)	1.38 (1.07–1.79)	0.013	1.37 (1.04–1.79)	0.023	
Former	246	162 (65.9)	376	279 (74.2)	1.23 (1.01–1.49)	0.037	1.72 (1.40–2.10)	1.61E-07	0.362
**Histology**									
Adenocarcinoma	230	133 (57.8)	314	192 (61.2)	1.11 (0.89–1.39)	0.354	1.65 (1.31–2.08)	2.63E-05	
Squamous cell carcinoma	110	66 (60.0)	165	118 (71.5)	1.22 (0.91–1.66)	0.189	1.55 (1.13–2.13)	0.007	
others	113	80 (70.8)	193	162 (83.9)	1.55 (1.18–2.02)	0.002	1.57 (1.19–2.08)	0.001	0.940
**Tumor stage**									
I-IIIA	247	101 (40.9)	381	199 (52.2)	1.42 (1.12–1.81)	0.004	1.30 (1.02–1.66)	0.035	
IIIB-IV	206	178 (86.4)	289	272 (94.1)	1.44 (1.19–1.74)	1.96E-04	1.55 (1.27–1.90)	1.47E-05	0.275
**Chemotherapy**									
No	250	119 (47.6)	354	223 (60.5)	1.49 (1.19–1.86)	4.54E-04	1.66 (1.31–2.09)	1.99E-05	
Yes	199	156 (78.4)	314	245 (78.0)	1.07 (0.88–1.31)	0.511	1.38 (1.12–1.70)	0.002	0.248
**Radiotherapy**									
No	293	151 (51.5)	434	275 (63.4)	1.39 (1.14–1.69)	0.001	1.45 (1.19–1.78)	2.86E-04	
Yes	156	124 (79.5)	234	193 (82.5)	1.12 (0.90–1.41)	0.318	1.74 (1.36–2.22)	8.78E-06	0.260
**Surgery**									
No	236	204 (86.4)	361	325 (90.0)	1.25 (1.05–1.49)	0.014	1.43 (1.19–1.71)	1.49E-04	
Yes	213	71 (33.3)	307	143 (46.6)	1.57 (1.18–2.09)	0.002	1.75 (1.31–2.35)	1.82E-04	0.250

Abbreviations: OS, overall survival; NSCLC, non-small cell lung cancer; PLCO, the Prostate, Lung, Colorectal and Ovarian Cancer Screening Trial; HR, hazards ratio.

[a]Risk genotypes included rs10794069 AG+GG, rs1732793 GA+AA, rs199731120 C/CA+CA/CA, rs35970494 T/TC+TC/TC, rs3806116 GT+TT.

[b]Adjusted by age, sex, smoking status, histology, tumor stage, chemotherapy, radiotherapy, and surgery when appropriate.

[c]*P* for heterogeneity in multivariate analyses.
